# Deep learning on lateral flow immunoassay for the analysis of detection data

**DOI:** 10.3389/fncom.2023.1091180

**Published:** 2023-01-26

**Authors:** Xinquan Liu, Kang Du, Si Lin, Yan Wang

**Affiliations:** ^1^School of Precision Instrument and Optoelectronics Engineering, Tianjin University, Tianjin, China; ^2^Tianjin Boomscience Technology Co., Ltd., Tianjin, China; ^3^Beijing Savant Biotechnology Co., Ltd., Beijing, China

**Keywords:** lateral flow immunoassay, data processing, point of care testing, deep learning, convolutional neural network, U-Net model

## Abstract

Lateral flow immunoassay (LFIA) is an important detection method *in vitro* diagnosis, which has been widely used in medical industry. It is difficult to analyze all peak shapes through classical methods due to the complexity of LFIA. Classical methods are generally some peak-finding methods, which cannot distinguish the difference between normal peak and interference or noise peak, and it is also difficult for them to find the weak peak. Here, a novel method based on deep learning was proposed, which can effectively solve these problems. The method had two steps. The first was to classify the data by a classification model and screen out double-peaks data, and second was to realize segmentation of the integral regions through an improved U-Net segmentation model. After training, the accuracy of the classification model for validation set was 99.59%, and using combined loss function (WBCE + DSC), intersection over union (IoU) value of segmentation model for validation set was 0.9680. This method was used in a hand-held fluorescence immunochromatography analyzer designed independently by our team. A Ferritin standard curve was created, and the T/C value correlated well with standard concentrations in the range of 0–500 ng/ml (*R*^2^ = 0.9986). The coefficients of variation (CVs) were ≤ 1.37%. The recovery rate ranged from 96.37 to 105.07%. Interference or noise peaks are the biggest obstacle in the use of hand-held instruments, and often lead to peak-finding errors. Due to the changeable and flexible use environment of hand-held devices, it is not convenient to provide any technical support. This method greatly reduced the failure rate of peak finding, which can reduce the customer’s need for instrument technical support. This study provided a new direction for the data-processing of point-of-care testing (POCT) instruments based on LFIA.

## 1. Introduction

*In vitro* diagnosis (IVD) generally refers to detecting targets in the blood, urine, sweat, saliva, tissue fluid, or tissue outside the body, and is mainly used to diagnose diseases, prevent infections, manage chronic diseases, track pathological changes, evaluate therapeutic effects, and other aspects of health care (Yang et al., 2021; Peng et al., 2022). Currently, the instruments used for IVD include biochemical, immunological, molecular, microbial, and blood diagnosis as well as point-of-care testing (POCT) (Haung and Ho, 1998; Xiao and Lin, 2015; Chen et al., 2017; Vila et al., 2017; Li et al., 2020; Liao et al., 2021). Compared with previous instruments, POCT has the characteristics of high speed, convenience, and low cost; therefore, it has received considerable attention from the medical industry (Singer et al., 2005; Damhorst et al., 2019).

Point-of-care testing is a patient-centered method for rapid sample detection using portable analytical instruments or simple reagents (Luppa et al., 2011; Florkowski et al., 2017). There are many kinds of POCT instruments, among which the lateral flow immunoassay (LFIA), based on paper-based and fluorescence detection technology, is increasingly being applied (Chen and Yang, 2015). It has the advantages of being cheap, lightweight, and easy to handle, and the fluorescence detection method can realize the quantitative detection of the sample. Both of them make LFIA highly competitive, especially for developing countries where budget is an important criterion, which is a good choice (Wu et al., 2018).

According to the published literature, LFIA technology has successfully realized the detection of biomarkers in many fields. Our research group combined many medical units using fluorescent microsphere labeling and immunochromatography technology to successfully detect COVID-19 and evaluated the analytical ability and clinical application of this technology (Zhang et al., 2020). Hu et al. (2016) developed a highly sensitive quantitative lateral flow analysis method for protein biomarkers using fluorescent nanospheres (FNs) as materials, which can be used to detect the concentration of CRP in the human body with a detection limit of 27.8 pM. Lee et al. developed a novel portable fluorescence sensor that integrates a lateral flow assay with quantum dots (Qdots) labeling and a mobile phone reader for the detection of Taenia solium T24H antibodies in human serum (Lee et al., 2019). Huang et al. (2020) used a double-antibody sandwich immunofluorescence method based on the combination of nano europium (EUNP) and lateral flow immunoassay (LFIA) to detect IL6 with a wide linear range (2–500 pg/ml) and high sensitivity (0.37 pg/ml) (Huang et al., 2020). Shao et al. (2017) used the double-antibody sandwich immunofluorescence method combined with the time-resolved immunofluorescence (TRFIA) and lateral flow immunoassay (LFIA) to detect human procalcitonin with high sensitivity (0.08 ng/ml). Gong et al. (2019) developed a miniaturized and portable UCNP-LFA platform that can be used to detect small molecules (ochratoxin A, OTA), heavy metal ions (Hg2+), bacteria (Salmonella, SE), nucleic acids (hepatitis B virus, HBV), and proteins (growth-stimulating expressed gene 2, ST-2).

**FIGURE 1:**
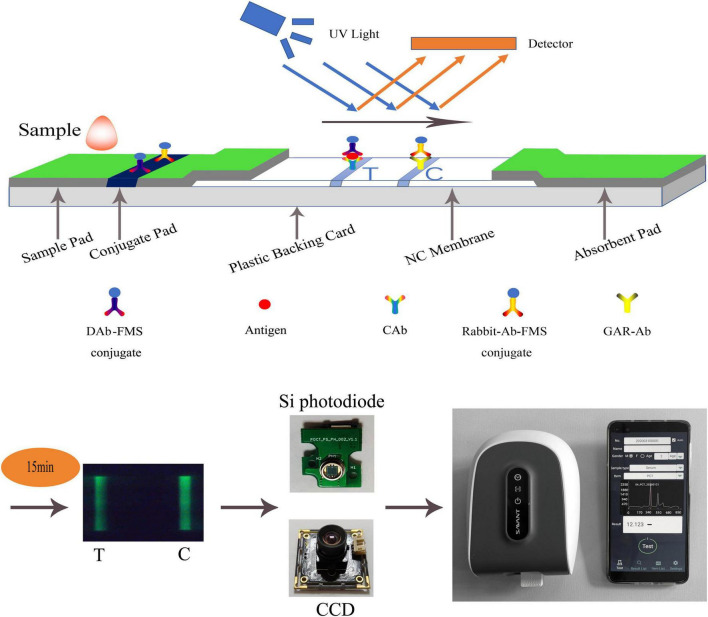
Schematic diagram of LFIA. A hand-held fluorescence immunoassay analyzer which was used to measure fluorescent intensity controlled by a mobile phone *via* Bluetooth. Its sensor can be CCD or Si photodiode.

As shown in Figure 1, there are two schemes of fluorescence detection technology for LFIA: a photoelectric scanning data acquisition platform based on Si photodiode, which is the current mainstream technology because of better performance, and a data acquisition platform based on CCD photography (Shao et al., 2019). The classical method of LFIA data processing is to obtain the C-/T- lines of the strip by peak-finding method. In this way, the normal peak and interference peak or noise peak cannot be distinguished, and wrong peak is easy to be regarded as normal peak, thus giving wrong detection result. These methods still perform poorly in effectively identifying weak and overlapping peaks while maintaining a low false-discovery rate. Qin et al. (2020) used a U-Net neural network, a variant of the convolutional neural network (CNN), to achieve the region of interest (ROI) containing T-/C-lines of test strips, and which was only used for CCD photography. In this study, we proposed a novel data processing method, which can be applied to both CCD photography and photoelectric scanning data acquisition platform. When applied to CCD photography, it only needed to convert the data to one dimension, which can be done by averaging the same row pixels parallel to the fluorescent band. This method greatly reduced the failure rate of peak finding, which can reduce the customer’s need for instrument technical support, and provided a new direction for the data processing of POCT instruments based on LFIA.

**FIGURE 2:**
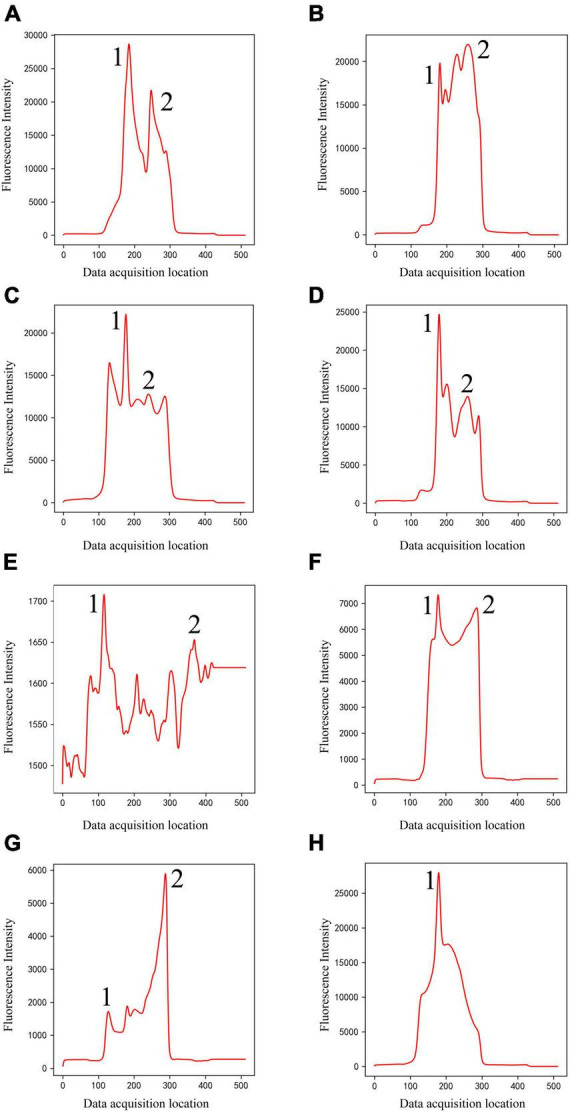
Noise (Class 1) of different shapes **(A–H)**. The classical methods will misjudge peak 1 as C-peak in panels **(A–G)**, and misjudge peak 2 as T-peak, resulting in incorrect detection results. They will also misjudge peak 1 as C-peak in panel **(H)**, and no T-peak can be found, resulting in a false concentration of 0.

Compared with the classical peak-finding method, method proposed in this study has the following advantages:

(1) Classical peak-finding methods combined with threshold-based techniques do not have the ability to identify peak shapes. They can only find local maxima according to certain rules, and cannot accurately identify certain noise signals as invalid data. For example, according to the setting rules in section “3.4. Comparison with classical methods,” they will misjudge peak 1 as C-peak in Figures 2A–G, and misjudge peak 2 as T-peak, resulting in incorrect detection results. They will also misjudge peak 1 as C-peak in Figure 2H, and no T-peak can be found, resulting in a false concentration of 0. In fact, all the data listed in Figure 2 were judged invalid by the technician. Due to the diversity of sample types and detection items, coupled with some problems in user operation, various invalid data could be generated. The classification model based on deep learning proposed in this study has ability to distinguish peak shape, and it can identify these invalid data as noise (class 1) or only T-peak (class 3), thus solving this problem well.

**FIGURE 3:**
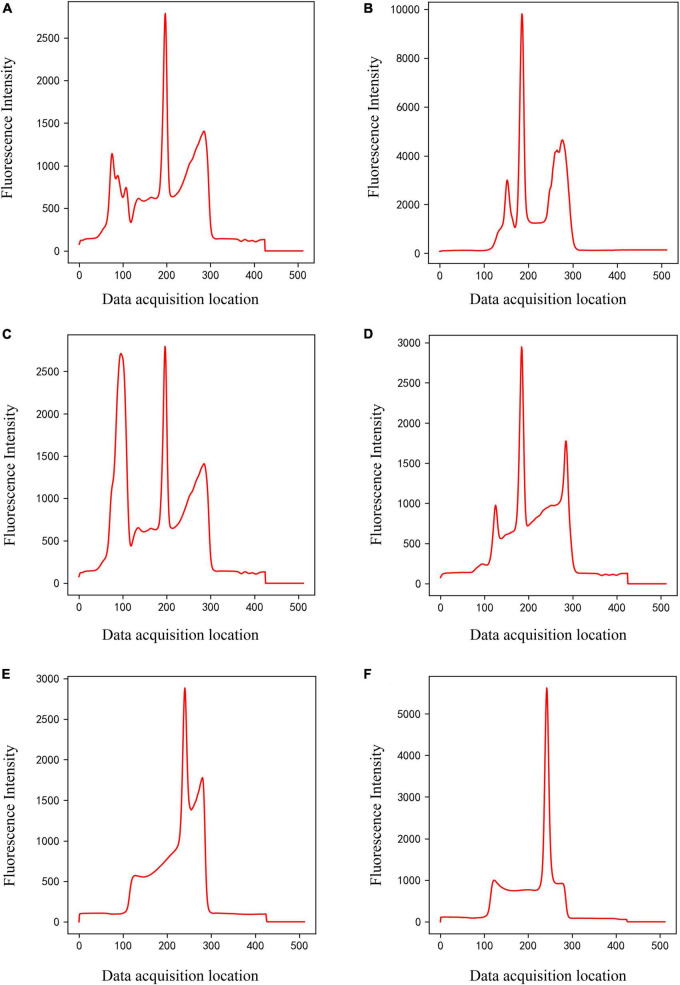
Panels **(A–D)** are only C-peak (Class 2) of different shapes, and panels **(E,F)** are only T-peak (Class 3) of different shapes.

**FIGURE 4:**
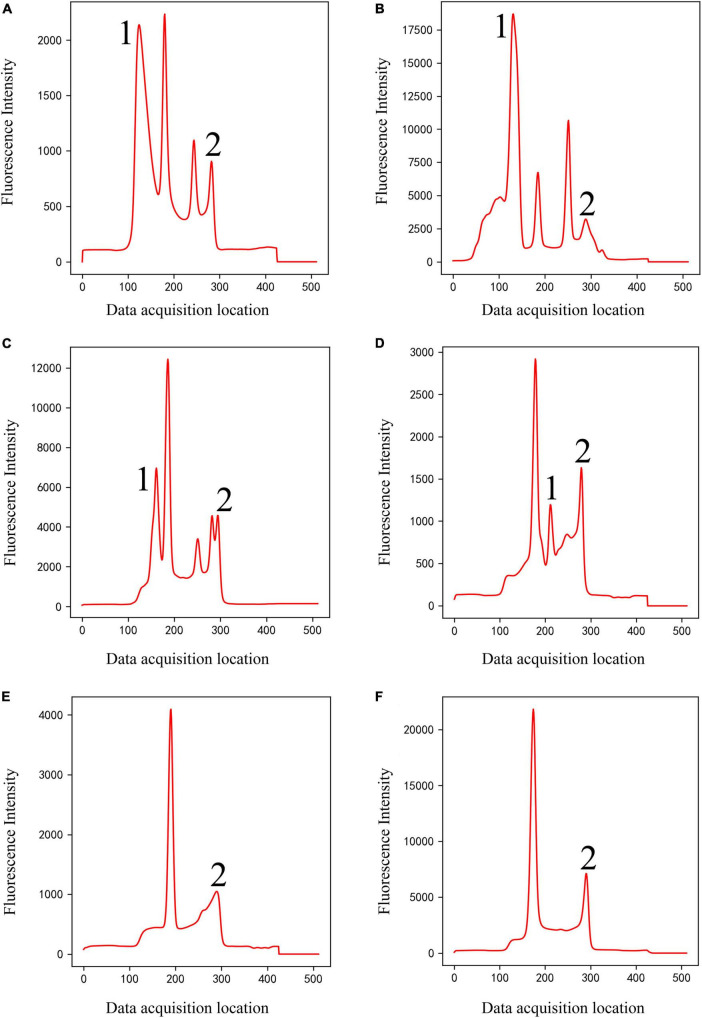
Double-peaks (Class 4) of different shapes **(A–F)**. Peaks 1 and 2 are interference peaks. The classic methods will misjudge peak 1 as C-peak in panel **(B)**, misjudge peak 2 as T-peak in panel **(C)**, and peak 1 or 2 as T-peak in panel **(D)**. They often fail to find T-peak and misjudge the tailing peak [peak 2 in panels **(E,F)**] as T-peak.

(2) Classical peak-finding methods cannot solve the problem of interference peaks, especially the interference peaks around weak T-peak, as shown in Figure 4. Interfering peaks may appear anywhere, to the left or right of valid peak. Classic peak-finding methods combined with threshold-based techniques, such as setting an interval range for the positions of C-peak and T-peak or setting a threshold for the height of C-peak, are not completely reliable. Because the positions of C- and T- peaks will change with assembly position of nitrocellulose membrane, insertion position of test strip, difference between different instruments, sampling speed and so on, errors will occur when the set range is exceeded. For example, the classic peak-finding methods will misjudge peak 1 as C-peak in Figure 4B, and misjudge peak 2 as T-peak in Figure 4C and peak 1 or 2 as T-peak in Figure 4D. In addition, the classic peak-finding methods perform poorly when looking for weak T-peak. They often fail to find T-peak and misjudge the tailing peak (peak 2 in Figures 4E, F) as T-peak. Similarly, the improved U-net segmentation model proposed in this study has ability to distinguish shape of peaks, which can solve this problem well.

(3) For classical methods, a minimum threshold is generally set for the height of C-peak. If the threshold is too small, accuracy will be greatly reduced due to presence of interference peaks or invalid data. If the threshold is too large, it will be unfavorable to process data with low height of C-peak in test strips of competition method. This is an unavoidable shortcoming of classical methods, but the method proposed in this study does not have this problem.
(4) Method proposed in this study can enhance its generalization ability by constantly learning new type data, but classical algorithm obviously does not have this ability. They are only some fixed peak-finding rules and threshold judgments, and cannot accurately identify some noise peaks similar to valid peaks. In particular, the noise data is ever-changing, and it is difficult for classical methods to be suitable for every new type of data.

## 2. Materials and methods

### 2.1. Materials

The data used for training, validation, and testing in this study were obtained from Beijing Savant Biotechnology Co., Ltd. These data are the result of testing a variety of items. The detection items mainly included human ferritin, vitamin D, D-dimer, and C-reactive protein and so on. The sample types mainly included whole blood, serum, and plasma.

### 2.2. Principle of LFIA

A double-antibody sandwich test strip with fluorescent microspheres (FMS) as the carrier was used to illustrate the detection principle of LFIA. The double-antibody sandwich structure is shown in Figure 1. The test strip was composed of a sample pad, conjugate pad, nitrocellulose membrane (NC membrane), absorbent pad, and plastic backing card. After the sample was dripped into the sample pad, it was subjected to immunochromatography under capillarity. The detection antibody-FMS (DAb-FMS) and rabbit IgG antibody-FMS (Rabbit-Ab-FMS) were placed on the conjugate pad. There are T and C lines on the NC membrane; the T line is coated with capture antibody (CAb), and the C line is coated with goat anti-rabbit IgG antibody (GAR-Ab). The absorbent pad causes liquid to flow *via* capillary action. The plastic backing card plays the role of fixing and supporting.

When the sample solution containing the analyte was added to the sample pad, it was laterally transferred along the NC membrane *via* capillary action. When the sample flowed through the conjugate pad, the Antigen in the sample reacted with DAb to form a DAb-FMS/Antigen complex. When the complex flows to the T line in the NC membrane, the Antigen and CAb on the T line are immunized to form a DAb-FMS/Antigen/CAb complex. Rabbit-Ab-FMS, which does not participate in the reaction, continues to flow forward to the C line and reacts with GAR-Ab.

Generally, the entire reaction process takes approximately 15 min. After immunochromatography is completed, the excitation light generated by the scanning mechanism irradiates the T and C lines, and fluorescence is generated. In the process of scanning the NC membrane, the fluorescence intensity produced at each point of the scan was recorded using a photodiode, and the peak data shown in Figure 1 were finally formed. The ratio of the fluorescence intensities of the two lines can be obtained by calculating the ratio of the peak areas of the T- and C-peaks. The concentration of the antigen detected in the sample was proportional to the T/C. By establishing a standard curve, the concentration of the antigen detected in the sample can be calculated.

### 2.3. Data augmentation

During the testing of clinical samples, four different peak shape data were obtained: noise (class 1), only C-peak (class 2), only T-peak (class 3), and double-peaks (C-peak and T-peak, class 4). Class 1 was generated by a fluorescence analyzer scanning the fouled NC membrane, whereas class 2 was generated by detecting the sample with a concentration of 0. However, the data of class 3 were very few, and were generally generated from the test strips with the disappearance of the C-peak. To better train the model, the C-peak of the double-peaks (class 4) was deleted and transformed into the background by a cubic spline interpolation method; thus, a large amount of data containing only the T-peak was generated manually.

### 2.4. Label annotation

To train the model, a large amount of labeled data is required. Data annotation is a complex process, and the quality of the annotation directly affects the results of the model training. This method includes two steps corresponding to a classification and a segmentation model, and the training data of the two models must be annotated separately. The labeled dataset was randomly divided into training and verification sets.

The entire dataset for the classification model includes approximately 4,100 detection data, including four types of peak shapes, namely, noise, only C-peak, only T-peak, and double-peaks. These four types of data were encoded according to one-hot, which were noise (class 1), only C-peak (class 2), only T-peak (class 3), and double-peaks (class 4), as shown in Figures 2–4. There were approximately 900 data for noise (class 1), 900 for only C-peak (class 2), 900 for only T-peak (class 3), and 1,400 for double-peaks (class 4). The peak shape of the detection data is particularly complex and diverse, and only a few typical ones are selected for display here.

The dataset of the segmentation model includes approximately 1,400 pieces of detection data, that is, all the data of class 4. In this study, based on the Python language, software was designed to annotate the integral regions of the T-peak and C-peak, and the integral regions of the C-peak and T-peak of 1,400 fluorescence detection data were annotated.

### 2.5. Network architecture

Convolutional neural network is an artificial neural network specially designed to process data such as images or videos. It generally has three layers, namely, convolution, pooling and full connection layer. In the convolution layer, input samples are convolved with kernel, and the discrete convolution function is defined as:


$$\left(f*g\right)\,\left(x\right)=\sum_{\tau }f\,\left(\tau \right)\cdot g\,\left(x-\tau \right)$$

where *f* and *g* are two functions.

Pooling is used to extract high-dimensional features, and the most commonly used ones are maximum and average pooling. In a fully connected layer, all neurons in the current layer are interconnected with every neuron in the next layer.

**FIGURE 5:**
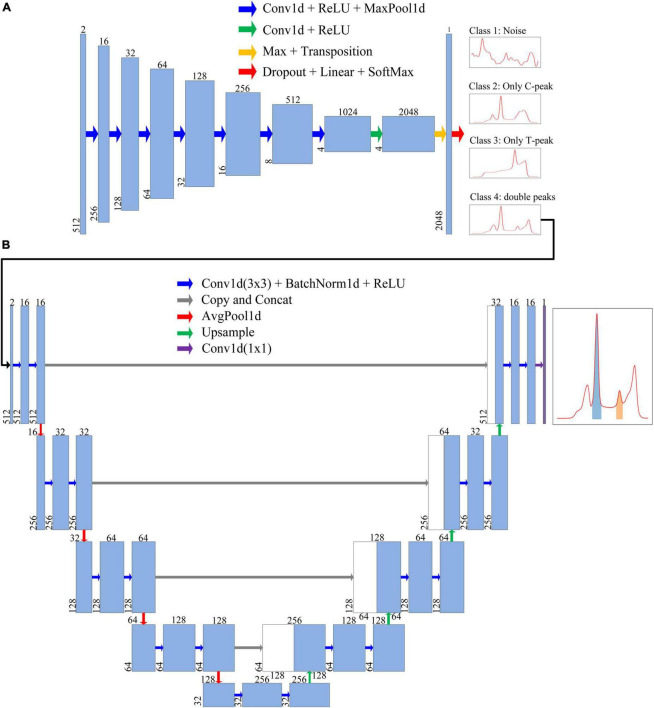
Each blue box represents a feature map of a layer. Number above the blue box is the number of channels, whereas number in lower left corner is the number of data points. The arrows represent different operations. **(A)** Neural network architecture of the classification model. The first blue box represents the format of input data. After being processed by the classification model, the input data were finally classified into four classes, namely, Class 1 (Nosie), Class 2 (Only C-peak), Class 3 (Only T-peak), and Class 4 (double-peaks). **(B)** Neural network architecture of segmentation model, through which the ROI of test strip containing T-/C- peak can be extracted and obtained.

As shown in Figure 5, the entire data-processing flow consists of two steps. First, a classification model was used to classify the input data. Second, after analyzing the input data, if the output result was class 4 (double-peaks), the data were imported into the next segmentation model to realize the data segmentation of the C-peak and T-peak areas.

The input of the classification model had two channels. Because the fluorescent signal has strong background noise, we subtracted the background and then normalized it as the first channel. It was achieved by the following formula.


$$Y_{1}=\frac{X-x_{m\,i\,n}}{x_{m\,a\,x}-x_{m\,i\,n}}$$

where *Y_1_* is the first channel, *X* is raw input data, *x*_*min*_ is minimum value, and *x*_*max*_ is maximum value of raw data. In order to make the model learn the peak shape rather than intensity, we performed a logarithmic operation on the signal which was deducted background as the second channel. It was achieved by the following formula.


$$Y_{2}=\frac{l\,o\,g_{10}\,\left(X-x_{m\,i\,n}\right)}{l\,o\,g_{10}\,\left(x_{m\,a\,x}-x_{m\,i\,n}\right)}$$

where *Y_2_* is the second channel, *X* is raw input data, *x*_*min*_ is minimum value, and *x*_*max*_ is maximum value of raw data.

The network architecture of the classification model is illustrated in Figure 5A. The entire network architecture consisted of 10 layers; the first seven layers were conv1d + ReLU + MaxPool1d (Acharya et al., 2017; Gu et al., 2018; Zhang et al., 2019), and the input data were extracted into four features of high-dimensional 1,024 channels. The eighth layer extended the number of channels to 2,048. Next, Max + Transposition was used to extract the maximum value from the four high dimension features (Gu et al., 2018). To improve the accuracy of classification, we used dropout layer before fully connected layers. The last layer (Dropout + Fully-connected + SoftMax) classified the data into one of four classes (Srivastava et al., 2014).

We designed an improved U-Net segmentation model with reference to the classic U-Net model (Ronneberger et al., 2015); the network architecture is shown in Figure 5B. We changed the input data into two channels. This model had four parts: input unit, encoding structure, decoding structure, and output unit (Ronneberger et al., 2015; Oh et al., 2019; Wang et al., 2021; Zheng et al., 2021; Zunair and Ben Hamza, 2021). The encoding structure used four units to reduce the dimensions, and the number of feature maps was increased gradually. In order to reduce training time, we added batch normalization after each convolution (Melnikov et al., 2020). In the decoding structure, each step was symmetrical with the encoding part to recover data. The upsampling section allowed the network to propagate the context information to a higher-resolution layer. In the last layer, the discrimination of whether each point in fluorescence data belonged to an integral region was realized.

### 2.6. Loss function

The classification model classified the data into one of four classes, which is a problem of four classes. Multi-classification neural networks generally use cross-entropy loss as a loss function. The mathematical expression of this loss function in the program is:


$$L_{C\,E}=-\frac{1}{M}\,\sum_{j=1}^{M}\sum_{i=1}^{C}y_{i\,j}\,l\,o\,g\,o_{i\,j}$$

where *M* is the batch size, *C* is the total number of classes (four), *y*_*ij*_ is the real label, and *o*_*ij*_ is the predictive output.

The Dice coefficient (also known as the Dice score or DSC) is a function of the set similarity measurement, which is usually used to calculate the similarity between two sets (Saeedizadeh et al., 2021), with values ranging from 0 to 1. Here, it was used to measure the overlap between the ground-truth and predicted masks, where 0 indicates no overlap and 1 indicates complete overlap.


$$D\,S\,C\,\left(A,B\right)=\frac{2\,\left|A\cap B\right|}{\left|A\right|+\left|B\right|}$$

where *A* and *B* denote the predicted and ground-truth masks.

To minimize the loss function, we used the 1-DSC as the final loss function. The mathematical expression of this loss function in the program is:


$$L_{D\,S\,C}=1-\frac{1}{M}\,\sum_{j=1}^{M}\frac{2\,\sum_{i=1}^{N}y_{i\,j}\,o_{i\,j}}{\sum_{i=1}^{N}y_{i\,j}+\sum_{i=1}^{N}o_{i\,j}}$$

where *M* is the batch size, *N* is the number of sample data, *y*_*ij*_ is the ground-truth mask, and *o*_*ij*_ is the predictive mask.

For unbalanced sample data, weighted binary cross entropy can be used as the training loss function. Therefore, compared with the standard cross-entropy loss, better results can be obtained when the number of positive and negative points is unbalanced (Zhu et al., 2019). The mathematical expression of this loss function in the program is:


$$L_{W\,B\,C\,E}=-\frac{1}{M\times N}\,\sum_{j=1}^{M}\sum_{i=1}^{N}\left(w_{1}\,y_{i\,j}\,l\,o\,g\,o_{i\,j}+w_{0}\,\left(1-y_{i\,j}\right)\,l\,o\,g\,\left(1-o_{i\,j}\right)\right)$$

where *M* is the batch size, *N* is the number of sample data, *y*_*ij*_ is the ground-truth mask, and *o*_*ij*_ is the predictive mask. *w_1_* and *w_0_* correspond to the weights labeled 1 and 0, respectively.

In this study, the mathematical expression for the weight parameter *w_c_* is:


$$w_{c}=\frac{N-N_{c}}{N}$$

where *N* represents the total number of data points for each sample and *N_c_* represents the number of data points in class *c*.

### 2.7. Model hyper-parameters of models

After labeling the data, we trained the model. The classification and segmentation models were trained separately. The training parameters of the classification and segmentation model are listed in Table 1.

**TABLE 1:** Important parameters used in two models training.

Network parameters	Classification model	Segmentation model
Batch size	8	8
Epoch	30	100
Activation function	ReLU	ReLU
Padding mode	MaxPool	AvgPool
Pooling size	2	2
Optimizer	Adam	Adam
Learning rate	0.001	0.001
Convolution kernel	3	3
Upsample	–	Nearest
Input size	512 × 2	512 × 2

## 3. Results

### 3.1. Evaluation metrics of models

Accuracy, which is the proportion of correctly predicted samples to the total number of samples, is generally used as the evaluation metric of a multi-classification model. The mathematical expression of accuracy in the program is:


$$Accuracy\left(y,o\right)=\frac{1}{M}\sum_{i=1}^{M}1\left(o_{i}=y_{i}\right)$$

where *M* denotes the batch size, *y_i_* denotes the real label, and *o*_*i*_ is the predictive output.

The intersection over union (IoU), also known as the Jaccard index, calculates the ratio of the intersection and union of the ground-truth and predicted segmentation masks (Saeedizadeh et al., 2021). It can be used to measure the similarity between the ground-truth and predicted segmentation masks; the higher the similarity, the higher the value.


$$I\,o\,U\,\left(A,B\right)=\frac{\left|A\cap B\right|}{\left|A\cup B\right|}$$

where *A* and *B* denote the predicted and ground-truth masks. The mathematical expression of IoU in the program is:


$$I\,o\,U\,\left(y,o\right)=\frac{1}{M}\,\sum_{j=1}^{M}\frac{\sum_{i=1}^{N}y_{i\,j}\,o_{i\,j}}{\sum_{i=1}^{N}y_{i\,j}+\sum_{i=1}^{N}o_{i\,j}-\sum_{i=1}^{N}y_{i\,j}\,o_{i\,j}}$$

where *M* is the batch size, *N* is the number of sample data, *y*_*ij*_ is the ground-truth mask, and *o*_*ij*_ is the predictive mask.

### 3.2. Model hyper-parameters optimization of segmentation model

Both the weight coefficients of the weighted binary cross-entropy and cut-off threshold have a certain influence on the performance of the model. To obtain appropriate weights and cut-off thresholds, this study conducted cross experiments on weights and cut-off thresholds. As presented in Table 2, when *w*_0_ : *w*_1_ = 0.6 : 0.4 and the cut-off threshold = 0.6, the IoU achieved a maximum value of 0.9680. The other parameters used during the training are listed in Table 1.

**TABLE 2:** Cross experiments result on weights of the weighted binary cross entropy and cut-off threshold.

w_0_:w_1_	IoU (Cut-off = 0.3)	IoU (Cut-off = 0.4)	IoU (Cut-off = 0.5)	IoU (Cut-off = 0.6)	IoU (Cut-off = 0.7)
0.3:0.7	0.9668	0.9652	0.9665	0.9677	0.9660
0.4:0.6	0.9674	0.9657	0.9658	0.9674	0.9674
0.5:0.5	0.9668	0.9674	0.9674	0.9665	0.9670
0.6:0.4	0.9677	0.9676	0.9666	0.9680	0.9674
0.7:0.3	0.9668	0.9674	0.9652	0.9669	0.9654

When w_0_:w_1_ = 0.6:0.4 and the cut-off threshold = 0.6, the IoU achieved a maximum value of 0.9680.

We also compared the three loss functions of WBCE, DSC, and WBCE + DSC. When the other conditions were the same, the combined loss function (WBCE + DSC) was used to obtain the maximum IoU, as illustrated in Table 3.

**TABLE 3:** Overall performance with different loss functions, *w*_0_:*w*_1_ = 0.6 : 0.4 and cut-off threshold = 0.6.

Loss	w_0_:w_1_	Cut-off	IoU
WBCE	0.6:0.4	0.6	0.9652
DSC			0.9586
WBCE + DSC			0.9680

### 3.3. Training convergence analysis of models

**FIGURE 6:**
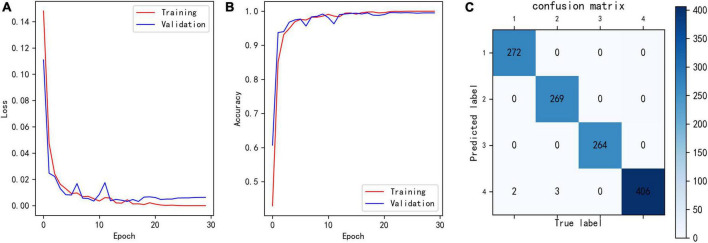
**(A)** Loss of classification model during training. **(B)** Training and validation accuracy of classification model during training. **(C)** Confusion matrix showing the result of trained classification model for validation set. The row number reflects the predicted label, and column number reflects the true label.

Figure 6A shows the loss curves of different epochs during the classification model training process, and Figure 6B shows the accuracy of the training and validation sets corresponding to different epochs. The maximum accuracy of the model validation set was 99.59%.

To analyze which samples were misclassified, we built confusion matrix. As in Figure 6C, only five samples were misclassified, two class 1 and three class 2 data were misclassified as class 4. These five samples had the characteristics of two different classes, which leaded to misclassification. In general, such samples are rare.

**FIGURE 7:**
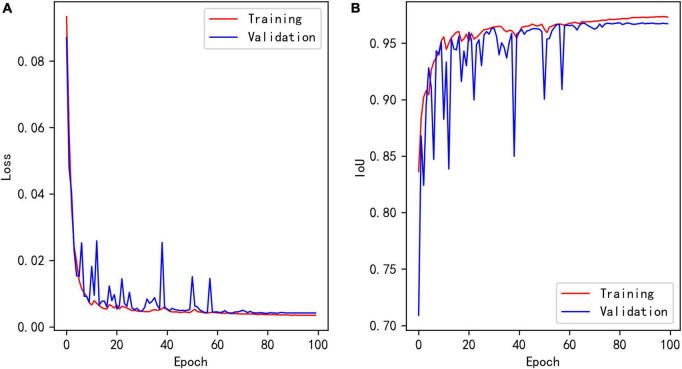
**(A)** Loss of segmentation model during training. **(B)** Training and validation IoU of segmentation model during training.

Figure 7A shows the loss curves of different epochs during the segmentation model training process, and Figure 7B shows the IoU of the training and validation sets corresponding to different epochs. The maximum IoU of the model validation set was 0.9680.

### 3.4. Comparison with classical methods

There are many types of peak detection methods, such as the direct peak location, Fourier transform, cumulative sum derivative, curve fitting, devolution, and wavelet transform (CWT) methods (Deng et al., 2021). The direct peak location according to the properties of peak and continuous wavelet transform are two classical methods in traditional methods. The principle of direct peak location is to find out all the local maxima of the signal through the simple comparison method, and then select the subset of these peaks according to the specified peak properties. The method principle of CWT is that the signal is first transformed by CWT in certain scales, and then the ridges are found in the CWT matrix. The positions of these ridges correspond to the positions of all peaks (Du et al., 2006). Using the verification set, method proposed in this paper was compared with the two traditional methods. These two methods have been implemented in SciPy library based on Python, so we directly used the related functions (find_peaks() and find_peaks_cwt()) in SciPy library.

Classical peak-finding methods can only find the local maxima of the signal, and do not have the ability to classify the signal. Here, after obtaining the local maxima through the classical methods, some subsequent processing steps were adopted to make it have the classification ability, and then compared with the classification model proposed in this study. These subsequent processing steps are as follows:

(1) According to the characteristics of the strip, the data of 512 sample points are divided into C peak region (0–220) and T peak region (221–511).
(2) Judge whether there are local maxima in the C peak region (0–220), and if so, take the maximal local maximum as the C peak. Judge whether the height of the C peak is greater than 1,000, and if it is greater than 1,000, it is considered to be an effective C peak (according to the characteristics of the strip, the height of the C peak is usually greater than 1,000).
(3) Judge whether there are local maxima in the T peak region (0–220), and if so, take the maximal local maximum as the T peak.
(4) According to the results of (2) and (3), the signal is classified to noise (class 1), only C-peak (class 2), only T-peak (class 3), and or double-peaks (class 4).

The comparison results are shown in Table 4. It can be seen that the performance of the two classical methods is similar in term of accuracy, one is 80.10%, the other is 80.76%. Accuracy of the method proposed in this study is 99.59%, which is much better than classical methods.

**TABLE 4:** Comparison of classical peak-finding methods and proposed method performance in terms of accuracy, IoU, dice, recall and precision.

Method	Accuracy	IoU	Dice	Recall	Precision
Direct peak location	80.10%	0.7753	0.8509	0.8801	0.8391
CWT	80.76%	0.7597	0.8423	0.8510	0.8541
Our method	99.59%	0.9680	0.9836	0.9857	0.9821

For two classical methods, the function of peak_widths() in the SciPy library can be used to identify the integral region. Compared with the segmentation method in this study in terms of IoU, Dice, Recall and Precision. The results are shown in Table 4. As can be seen from the table, no matter which evaluation term it is, the method proposed in this paper is much better than two classical methods.

### 3.5. Test of the method

The method proposed in this study was tested using instrument test data. First, the ability of the segmentation model to segment various peak shapes was tested. Next, three most important indicators (standard curve, repeatability, and recovery) were tested.

**FIGURE 8:**
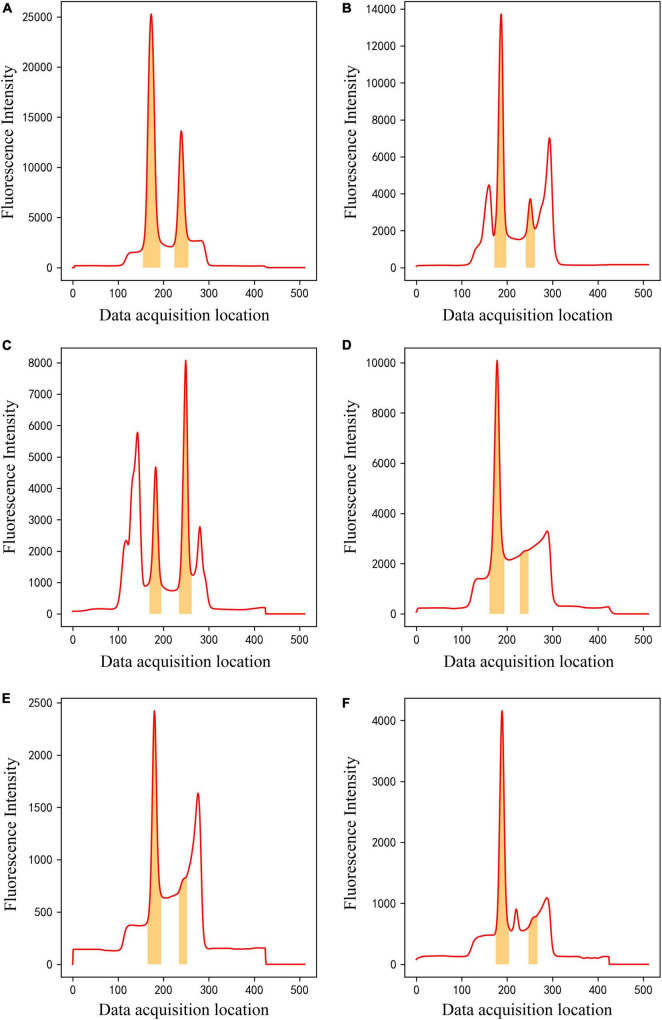
The results of ROI extraction by segmentation model on different kinds of data. **(A)** Normal peak data, **(B,C)** overlapping and interference peak data, **(D–F)** Weak T-peak with baseline drift, tailing or interference peak data.

After training, the method can classify raw input data into one of four classes and perform data segmentation on data belonging to Class 4. The segmentation model could effectively segment C- and T-peak regions from fluorescence intensity of 512 data points. Figure 8 shows examples of data segmentation results for some typical peak shapes, where the orange shaded areas are segmented C- and T-peak regions. Figure 8A shows segmentation of the normal peak shape, and C -and T-peak regions were accurately extracted and obtained. Figures 8B, C show that in the presence of overlapping and interference peaks, C- and T-peaks can be accurately segmented. Figures 8D–F show the segmentation results for weak T-peak with baseline drift, tailing or interference peak. As shown in the figure, baseline drift, tailing and interference peaks did not affect accurate segmentation of the data; the detection of weak T-peak region is also excellent. After data were imported into the segmentation model, they were first normalized. The network model only focused on learning the shape of entire data set and did not learn the value of fluorescence intensity. The experimental results indicate that it can meet the requirements of LFIA for data processing.

**FIGURE 9:**
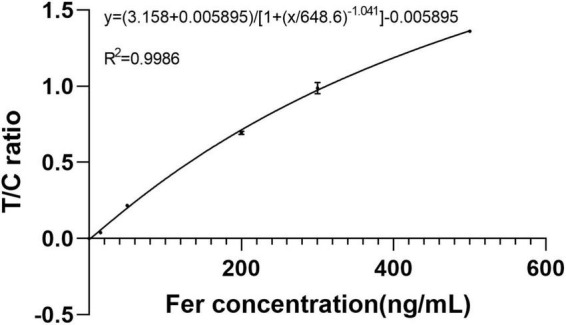
Four parameter fitting line for ferritin detection in the range of 0–500 ng/ml.

The method was tested using Ferritin. A standard curve was established using a range of concentrations (0, 15, 50, 200, 300, and 500 ng/ml) of the standards. Each concentration of the standard was tested three times using test strips. The detection data were processed using proposed method. First, data were classified, then segmented, and finally, the segmented regions were integrated and T/C was calculated. The method accurately classified the detection data of 0 ng/ml as class 2 (only C-peak), and the corresponding T/C values were 0. The remaining data were classified as class 4 (double-peaks) and then segmented. Using T/C as the ordinate and concentration as the abscissa, a standard curve was established using four parameters, as shown in Figure 9. It can be observed that the T/C and concentration have a good correlation with a correlation coefficient of 0.9986. This shows that the method is effective in dealing with LFIA data.

Three concentrations (20, 220, and 400 ng/ml) of the reference standards were tested for repeatability using the same batch of test strips. Each concentration was tested 10 times, and the CV values were calculated. The data were processed using the method described in this study. The data for all the three concentrations were classified as class 4 (double-peaks). The data were segmented, and concentrations were calculated; the results are listed in Table 5. It can be observed that the CV values of three concentrations are all good, and the maximum does not exceed 1.37%. This shows that the stability of the method is good.

**TABLE 5:** Precision results of ferritin test strips.

Mean (ng/ml)	SD	CV (%)
17.561	0.240	1.37
212.541	1.274	0.60
369.034	1.401	0.38

Recovery was tested using samples of three concentrations (40, 100, and 150 ng/ml). Each sample was tested thrice. The method in this study was used to process the data, and all the data were classified as class 4 (double-peaks); the results are listed in Table 6. The calculated recovery rates were 105.07, 96.37, and 99.67%, respectively. This shows that concentration calculated by the method is very accurate.

**TABLE 6:** Recovery rates results of Ferritin test strips.

Concentration (ng/ml)	Mean (ng/ml)	Recovery rate (%)
40	42.030	105.07
100	96.371	96.37
150	149.502	99.67

## 4. Discussion

Because POCT instruments based on LFIA detection technology are used in a variety of situations and there are many different types of samples, the peak shape of the test data is complex. It is difficult for classical peak-finding methods to deal with all peak shapes. The data-processing method proposed in this study has several advantages.

First, through a classification network, the peak types were classified into four classes, and the peak types that needed to be calculated for the concentration were screened. In this manner, the data processing difficulty of the segmentation model is reduced, and the model can easily achieve better performance. Second, an improved U-Net-based segmentation model directly identifies the integration regions, replacing the operations of the peak finding, peak start and end location in the classical method, which makes the data processing process more accurate and convenient. It is very difficult to determine the starting point and ending point of the peak accurately by the traditional method. Our segmentation model can easily solve this problem. Third, through experiments, it was found that this U-Net -based segmentation method also performs well in effectively identifying weak and trailing peaks. Forth, the classical peak-finding methods can only find the local maxima of the signal, and do not have the ability to classify the signal. In this case, it is difficult to distinguish the noise peak from the effective peak. Our classification model has perfectly solved this problem.

The method was applied to the hand-held immunofluorescence analyzer developed by ourselves and good results were obtained. Interference peaks are the biggest obstacle in the use of hand-held instruments, and often lead to peak-finding errors. The use environment of hand-held instruments is flexible and changeable, which makes it inconvenient to provide technical support. This method greatly reduced the failure rate of peak finding, which can reduce the customer’s need for instrument technical support. This is a great advantage for hand-held instruments sold in large quantities.

## 5. Conclusion

In this study, a deep-learning-based LFIA photoelectric scanning data-processing method was proposed. The entire method had two steps. The first step was to build a CNN classification model to classify the LFIA peak shape and screen out the data required to calculate the concentration. The second step was to build an improved 1D U-Net segmentation model to achieve the segmentation of C- and T-peak integration regions for data containing double-peaks and then perform calculations such as T/C and concentration. A large amount of experimental data were used to train the two models. The accuracy of classification model on validation set was 99.59% and the IoU of segmentation model on validation set was 0.9680. Using the data-processing method, a standard curve was established for Ferritin, and the CV and recovery rate, the two most relevant indicators in clinical testing, were tested. The CV values corresponding to the three concentrations of 20, 220, and 400 ng/ml were 1.37, 0.60, and 0.38%, respectively. The recovery rates corresponding to the three concentrations of 40, 100, and 150 ng/ml were 105.07, 96.37, and 99.67%, respectively. These experimental results show that the data-processing method proposed in this study can be used for the processing of LFIA photoelectric scanning data, and the obtained results are accurate and reliable, which proposes a new direction for POCT instrument data processing.

## Data availability statement

The raw data supporting the conclusions of this article will be made available by the authors, without undue reservation.

## Author contributions

XL and YW involved in the conception and research design. XL, KD, and SL collected the data and annotated the data. XL performed the statistical analysis and wrote the manuscript. XL, KD, SL, and YW revised it for publication. All authors contributed to the article and approved the submitted version.
